# Quantitative trait loci influencing pentacyclic triterpene composition in apple fruit peel

**DOI:** 10.1038/s41598-019-55070-5

**Published:** 2019-12-06

**Authors:** John T. Christeller, Tony K. McGhie, Jason W. Johnston, Bridie Carr, David Chagné

**Affiliations:** 1 The New Zealand Institute for Plant and Food Research Ltd (Plant & Food Research), Palmerston North Research Centre, Palmerston North, New Zealand; 2 Plant & Food Research, Havelock North, New Zealand; 3 Department of Agriculture and Fisheries, Maroochy Research Station, 47 Mayers Road, Nambour, QLD 4560 Australia

**Keywords:** Plant genetics, Plant molecular biology

## Abstract

The chemical composition of pentacyclic triterpenes was analysed using a ‘Royal Gala’ x ‘Granny Smith’ segregating population in 2013 and 2015, using apple peels extracted from mature fruit at harvest and after 12 weeks of cold storage. In 2013, 20 compound isoforms from nine unique compound classes were measured for both treatments. In 2015, 20 and 17 compound isoforms from eight unique compound classes were measured at harvest and after cold storage, respectively. In total, 68 quantitative trait loci (QTLs) were detected on 13 linkage groups (LG). Thirty two and 36 QTLs were detected for compounds measured at harvest and after cold storage, respectively. The apple chromosomes with the most QTLs were LG3, LG5, LG9 and LG17. The largest effect QTL was for trihydroxy-urs-12-ene-28-oic acid, located on LG5; this was measured in 2015 after storage, and was inherited from the ‘Royal Gala’ parent (24.9% of the phenotypic variation explained).

## Introduction

Fruit tree breeding has typically focussed on key horticultural characters including yield, pest and disease resistance and fruit qualities such as attractiveness and taste. Nevertheless, consumers are increasingly purchasing fruit for their perceived health promoting attributes. For example, purchase may be influenced by the fruit composition in dietary molecules such as fibre, micronutrient and antioxidant compounds. Some evidence is accumulating about phytochemical compounds characterised *in vitro* and *in vivo* for their effects on human health. One type of such compounds, the triterpenic acids such as ursolic acid (UA)^1^, oleanolic acid (OA) and betulinic acid (BA), has been suggested to have a broad range of effects on health characteristics. The primary literature, spanning *in silico* experiments, cell studies and small animal research, on the health benefits is voluminous and will not be summarised here. The subject is widely reviewed and the reader is directed to the following references as a starting point^2–12^, wherein the potential for conjugated and non-conjugated pentacyclic triterpenes as anti-inflammatory^7^, anti-microbial, anti-fungal and anti-protozoal agents, as well as inhibiting initiation, promotion, and metastasis of cancer^4,7,9^, anabolic effects on skeletal muscles, the ability to suppress bone density loss leading to osteoporosis, and health beneficial effects against cardiovascular disease, asthma and pulmonary dysfunction, diabetes, and obesity, is assessed. While the bulk of these experimental data have been obtained using UA, lupeol and saponins on account of their ready availability, they does not preclude the efficacy of the multitude of closely related compounds present in plant tissues.

The pathway for the biosynthesis of pentacyclic triterpenes is well established. The dedicated pathway starts with the condensation of dimethylallyl diphosphate and isopentenyl diphosphate to produce farnesyl diphosphate, two molecules of which lead to squalene and hence oxidation to 2, 3-oxidosqualene. These reactions are carried out by the enzymes farnesyl diphosphate synthase, squalene synthase and squalene epoxidase. 2, 3-Oxidosqualene is the key intermediate and the substrate for a range of monofunctional and multifunctional oxidosqualene cyclases, in one conformation leading to lanosterol, cycloartenol and hence to brassinosteroids and phytosterols, and in a second conformation to germanicol, lupeol, α-amyrin, β-amyrin and other triterpenes^13^. The molecular control of cyclic triterpene synthesis in apple was attributed to triterpene synthases (oxidosqualene synthases; OSCs)^13,14^. The triterpenic acids produced oxidatively from these four compounds by cytochrome P450 enzymes are morolic acid, BA, UA and OA respectively. The immense range of cyclic triterpenes (>20,000 have been identified to date) are then produced by further oxidative embroidery by P450 enzymes to produce dihyroxylated, trihydroxylated, and keto derivatives^15–18^ as well as glycosylation to produce saponins and esterification with hydroxycinnamic acids^19,20^.

In fruit, the bulk of the triterpenes are located in the waxy cuticle layer on the surface of the skin^1,11^. These authors report triterpene content of apple fruit at 0.28–0.34% of peel dry weight and 18–19.5% of peel wax extract. While the pharmaceutical properties continue to be researched extensively, the role of triterpenes in surface waxes of fruit has received scant attention. No specific role in reduction of water permeability has been suggested although a role as substrates for enhanced cytotoxic pharmaceuticals has been noted^11^. As such a role in insect and pathogen resistance could be anticipated and indeed negative effects on caterpillar growth by feeding on diets containing ursolic acid have been observed^21^.

Differences in triterpene composition have been measured across 109 apple cultivars^22^, including an in-depth study of differences between ‘Royal Gala’ and ‘Merton Russet’^19^, indicating potential genetic control. However, to date no knowledge exists about the genetic control and inheritance of such compounds. Here, we present a study about a specific class of compound with potential health properties, the pentacyclic triterpenes and map quantitative trait loci (QTLs) for it onto the apple genome.

## Methods

### Plant material and fruit treatment

The plant material consisted of 124 apple trees comprising a segregating cross of *Malus* x *domestica* ‘Royal Gala’ x ‘Granny Smith’ (RGxGS)^23^, including both parents. The fruit were collected from an orchard in Havelock North, New Zealand. All scions were grafted onto ‘Malling 9’ rootstock and planted 1.5 m apart in four rows in 2009.

In 2013 and 2015, ten and six apples from each tree were taken randomly at maturity, respectively. Maturity was assessed based on background colour change and starch clearance [3–4 units on a New Zealand industry generic 0–7 starch pattern index (SPI) scale^24^; and half of the fruit picked were shipped overnight to Palmerston North, New Zealand for immediate initial processing. The remaining fruit were stored for 12 weeks at 0.5 °C and then shipped to Palmerston North. This cold storage treatment was applied to induce even ripening between all fruit. All cold-stored fruit were then kept at 20 °C for seven days before being processed for chemical analysis, again to ensure even ripening. The fruit were manually peeled equatorially to remove approximately 80% of the surface of each fruit and approximately equal weights of peel from each apple were combined, frozen in liquid nitrogen, blended in a blender precooled with liquid nitrogen, and the consequent powder stored at −80 °C. Because of the variation in time to flowering and to subsequent maturity for this segregating population, this period was about 6 weeks. After all powdered samples had been accumulated, further processing to produce liquid samples for liquid chromatography–mass spectrometry (LC-MS) was carried out for randomly selected sets of powders. A measured weight of about 1 g of each powder was suspended by manually shaking in 5 mL ethanol (containing 1% formic acid) before rotating on a mechanical shaker for 1 h at room temperature. The samples were then centrifuged at 3000 rpm × 10 min (Eppendorf 5804 centrifuge) and 700 µL of each sample transferred to a 96-well microtitre plate and stored at −80 °C until analysed. In 2015, samples for LC-MS were produced by taking 750 µL of the ethanolic extract and adding 4 mL hexane and 1 mL water, shaking and centrifuging again as above; the upper phase was stored at −20 °C until analysis.

### Triterpene analysis by LC-MS

LC-MS grade acetonitrile and formic acid were purchased from Fischer Scientific; Ultrapure water was obtained from a Milli-Q Synthesis system (Millipore).

Stored sample extracts were diluted 2-fold with methanol before analysis by LC-MS. The liquid chromatography - high resolution accurate mass - mass spectrometry (LC-HRAM-MS) system used for analysis was composed of a Dionex Ultimate® 3000 Rapid Separation LC and a micrOTOF QII high resolution mass spectrometer (Bruker Daltonics, Bremen, Germany) fitted with an electrospray ion source. Two LC columns, joined by a low-volume connector, were used for separation of terpenoid compounds. Both columns were Zorbax SB-C18 150 × 2.1 mm, 1.8 µm (Agilent, Melbourne, Australia) and were maintained at 60 °C. The flow was 400 µL/min. The solvents were A = 100% acetonitrile and B = 0.2% formic acid. The solvent gradient was: 10%A, 90% B, 0–0.5 min; linear gradient to 100% A, 0.5–22 min; composition held at 100% A, 22–28 min; linear gradient to 10% A, 90% B, 28–28.2 min; to return to the initial conditions before another sample injection at 31 min. The injection volume for samples and standards was 1 μL. The micrOTOF QII parameters for polyphenolic analysis were: temperature 225 °C; drying N_2_ flow 6 L/min; nebulizer N_2_ 1.5 bar, endplate offset −500 V, mass range 100–1500 Da, acquired at a rate of 2 scans/s. Negative ion electrospray was used with a capillary voltage of +3500 V. Post-acquisition internal mass calibration used sodium formate clusters with the sodium formate delivered by a syringe pump at the start of each chromatographic analysis.

Compounds were identified using the accurate mass of the [M-H]^−^ ion and by reference to previous reports (McGhie *et al*., 2012). Peak areas were calculated from exact ion chromatograms (±10 mDa) for each molecular formula using QuantAnalysis (Bruker Daltonics, Bremen, Germany) software.

### Genetic map and QTL analysis

The genetic linkage map of ‘Royal Gala’ x ‘Granny Smith’ (RGxGS) was constructed using the International RosBREED SNP Consortium Apple single nucleotide polymorphism (SNP) array^25^ as published by Souleyre, Chagne, Chen, Tomes, Turner, Wang, Maddumage, Hunt, Winz, Wiedow, Hamiaux, Gardiner, Rowan and Atkinson^26^. The genetic map consists of 293 and 324 SNP markers for the ‘Royal Gala’ and ‘Granny Smith’ parents, and covers 904.9 and 1106.1 cM, for a density of one SNP every 3.08 and 3.41 cM in 17 linkage groups, respectively (Supplemental Material 1). QTL analysis was performed separately on the ‘Royal Gala’ and ‘Granny Smith’ parental maps using the compound concentration values extracted from each genotype as phenotypic data. The compound concentrations at harvest and after storage of the fruit were treated independently. The MapQTL® 5.0 software (www.kyazma.nl) was used to detect QTLs. The significance LOD thresholds at 90%, 95% and 99% genome-wide were calculated using 1,000 permutations. QTLs were detected using Interval Mapping (IM) using steps of 1 cM for each trait and parental map. The variance explained by the QTLs was extracted from MapQTL® 5.0. For non-normally distributed traits, the Kruskal-Wallis test was used for verifying QTLs.

### Data archiving

The genetic map data for the ‘Royal Gala’ x ‘Granny Smith’ population was previously published (cited).

## Results

### Segregation of chemical compounds in the ‘Royal Gala’ x ‘Granny Smith’ population

Metabolite composition was analysed using the RGxGS segregating population in 2013 and 2015 in apple peels from mature fruit at harvest and after 12 weeks cold storage. In 2013, 20 variables corresponded to pentacyclic triterpenes. In 2015, 196 metabolites in total were measured in the fruit using LC-HRAM-MS across genotypes/treatments (Supplemental Material 1). Metabolites were characterised using their measured accurate mass ([M-H]^−^) and the pentacyclic triterpenes selected. A total of 77 were quantified and kept for further statistical analysis (Supplemental Material 1). Nine, 41 and 27 variables have bimodal, skewed and normal distributions, respectively.

Multiple isoforms were identified for most classes of triterpene metabolites and were labelled with a one-letter suffix to indicate they are different. These isoforms have similar accurate masses but different retention times suggesting that they are structurally related isomers. The triterpene metabolites investigated here have a base core of urs-12-ene-28-oic acid with various numbers of additional hydroxy and keto groups. The isoforms most likely arise due to the variable position of the hydroxy and keto groups around the base ursine core. For example, for p-coumaroyloxy-hydroxy-urs-12-ene-28-oic acid three isoforms were detected in 2013.

**Figure 1:**
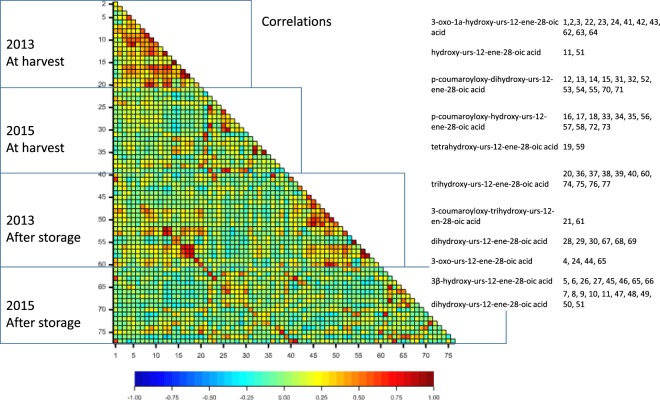
Phenotypic correlations between pentacyclic triterpenes in the ‘Royal Gala’ x ‘Granny Smith’ progeny over two years and two treatments. The correlation coefficients and probabilities are given in Supplemental Material 2 and shown as colours on the figure. Numbers corresponding to each compound are indicated.

In 2013, 20 compound isoforms were measured both after harvest and after cold storage. These 20 isoforms were from nine unique triterpene classes. The most concentrated compound in 2013 was p-coumaroyloxy-dihydroxy-urs-12-ene-28-oic acid for both treatments and the lowest concentrated compound was 3-oxo-urs-12-ene-28-oic acid for both treatments. In 2015, 20 and 17 compound isoforms were measured after harvest and cold storage, respectively. These 20 and 17 isoforms were from eight unique triterpene classes. The most concentrated compound in 2015 was dihydroxy-urs-12-ene-28-oic acid at harvest and 3β-hydroxy-urs-12-ene-28-oic acid after storage, and the lowest concentrated compound was trihydroxy-urs-12-ene-28-oic acid for both treatments. The correlation coefficients and probabilities between compounds are shown in Fig. 1 and Supplemental Material 2. For a given compound and year, phenotypic correlations between fruit at harvest and after storage ranged widely. Hydroxy-urs-12-ene-28-oic acid in 2013, 3-oxo-1a-hydroxy-urs-12-ene-28-oic acid in 2015 and 3β-hydroxy-urs-12-ene-28-oic acid (B isoform) in 2013 had correlation coefficients close to zero between harvest and after storage. Conversely, p-coumaroyloxy-hydroxy-urs-12-ene-28-oic acid (C isoform) and p-coumaroyloxy-dihydroxy-urs-12-ene-28-oic acid (A isoform) in 2013 had *r*^2^ > 0.75. Of 37 compounds detected both at harvest and after storage, concentration decreased, increased or remained stable in nine, 14 and 14 compounds, respectively (Supplemental Material 1).

### QTL mapping

QTL analysis was performed using 77 variables using the SNP-based linkage map of RGxGS. In total, 68 QTLs were detected on 13 linkage groups (LG) (Table 1) and were above a LOD score of 2.4, corresponding to a genome-wide significance of 90% calculated using a permutation test. Fifty-seven and 11 QTLs were detected for 2013 and 2015, respectively. Thirty-two and 36 QTLs were detected for compounds measured at harvest and after cold storage, respectively. The apple chromosomes with the most QTLs were LG3, LG5, LG9 and LG17. The largest effect QTL was for trihydroxy-urs-12-ene-28-oic acid on LG5, measured in 2015 after storage and inherited from the ‘Royal Gala’ parent (24.9% of the phenotypic variation explained). The robustness of QTLs is often verified as variables showing significant QTLs across years. The QTL hotspot on LG3 had a year stable QTL for trihydroxy-urs-12-ene-28-oic acid at harvest in both 2013 and 2015, inherited from the ‘Granny Smith’ parent. Of the QTLs detected on LG5, trihydroxy-urs-12-ene-28-oic acid and 3-oxo-hydroxy-urs-12-en-28-oic acid were stable across years and both were inherited from the ‘Royal Gala’ parent. A QTL for trihydroxy-urs-12-ene-28-oic acid found on LG16 of ‘Granny Smith’ was significant both in 2013 and 2015. All eight and eleven QTLs detected on LG9 and LG17, respectively, were from data collected in 2013.

**Table 1:** Quantitative trait loci detected for pentacyclic triterpene compounds in the ‘Royal Gala’ x ‘Granny Smith’ segregating population.

Compound name	Isoform	Year	Fruit sample assessed	Parent contributing the QTL allele	Chr	LOD	% variance explained by QTL	Closest marker to QTL peak
3-coumaroyloxy-dihydroxy-urs-12-en-28-oic acid	na	2015	Storage	Royal Gala	5	3.54	15.2	5_6078855
3-coumaroyloxy-trihydroxy-urs-12-en-28-oic acid	na	2015	Storage	Granny Smith	8	3.01	13.1	8_18192194
3-oxo-1a-hydroxy-urs-12-ene-28-oic acid	A	2013	harvest	Royal Gala	3	4.94	17	3_1143761
3-oxo-1a-hydroxy-urs-12-ene-28-oic acid	A	2013	storage	Granny Smith	3	4.2	14.4	3_7171067
3-oxo-1a-hydroxy-urs-12-ene-28-oic acid	A	2013	storage	Royal Gala	5	6.31	20.9	5_34448952
3-oxo-1a-hydroxy-urs-12-ene-28-oic acid	A	2013	harvest	Royal Gala	5	3.32	11.8	5_36487489
3-oxo-1a-hydroxy-urs-12-ene-28-oic acid	A	2013	harvest	Granny Smith	10	3.21	11.4	10_11942818
3-oxo-hydroxy-urs-12-en-28-oic acid	na	2015	Harvest	Royal Gala	5	3.36	12.4	5_36487489
3-oxo-hydroxy-urs-12-en-28-oic acid	na	2015	Storage	Royal Gala	5	6.02	24.4	5_36508128
3-oxo-hydroxy-urs-12-ene-28-oic acid	C	2013	storage	Granny Smith	3	2.5	8.9	3_34437600
3-oxo-hydroxy-urs-12-ene-28-oic acid	B	2013	storage	Royal Gala	5	2.48	8.8	5_21402981
3-oxo-hydroxy-urs-12-ene-28-oic acid	C	2013	storage	Royal Gala	5	3.78	13.1	5_15472298
3-oxo-hydroxy-urs-12-ene-28-oic acid	C	2013	harvest	Royal Gala	5	3.25	11.5	5_34448952
3-oxo-hydroxy-urs-12-ene-28-oic acid	C	2013	storage	Granny Smith	6	3.91	13.5	6_12007655
3-oxo-hydroxy-urs-12-ene-28-oic acid	B	2013	storage	Royal Gala	9	2.7	9.5	9_5964010
3-oxo-hydroxy-urs-12-ene-28-oic acid	C	2013	harvest	Royal Gala	17	3.72	13.1	17_21775385
3-oxo-hydroxy-urs-12-ene-28-oic acid	C	2013	storage	Royal Gala	17	3.01	10.6	17_8244579
3-oxo-urs-12-ene-28-oic acid	na	2015	Storage	Royal Gala	1	3.79	16.7	1_35422934
3-oxo-urs-12-ene-28-oic acid	A	2013	storage	Royal Gala	5	2.75	9.7	5_36487489
3?-hydroxy-urs-12-ene-28-oic acid	B	2013	storage	Royal Gala	6	2.63	9.3	6_1640609
3?-hydroxy-urs-12-ene-28-oic acid	B	2013	storage	Granny Smith	12	2.95	10.4	12_22208
dihydroxy-urs-12-ene-28-oic acid	A	2013	harvest	Royal Gala	4	3.53	12.5	4_19761986
dihydroxy-urs-12-ene-28-oic acid	A	2013	storage	Royal Gala	4	3.09	10.9	4_2768879
dihydroxy-urs-12-ene-28-oic acid	C	2013	storage	Royal Gala	5	3.71	12.9	5_10289990
dihydroxy-urs-12-ene-28-oic acid	C	2013	harvest	Royal Gala	5	4.67	16.2	5_17565719
dihydroxy-urs-12-ene-28-oic acid	D	2013	harvest	Royal Gala	5	5.4	18.4	5_17565719
dihydroxy-urs-12-ene-28-oic acid	C	2013	harvest	Granny Smith	7	2.79	10	7_13557557
dihydroxy-urs-12-ene-28-oic acid	D	2013	storage	Royal Gala	10	2.74	9.7	10_27656951
dihydroxy-urs-12-ene-28-oic acid	A	2013	storage	Granny Smith	13	2.53	9	13_38238894
dihydroxy-urs-12-ene-28-oic acid	C	2013	harvest	Granny Smith	13	2.88	10.3	13_6017316
dihydroxy-urs-12-ene-28-oic acid	D	2013	harvest	Granny Smith	13	2.72	9.8	13_6017316
dihydroxy-urs-12-ene-28-oic acid	D	2013	harvest	Royal Gala	16	2.69	9.6	16_14676540
dihydroxy-urs-12-ene-28-oic acid	C	2013	harvest	Royal Gala	17	2.88	10.3	17_21775385
dihydroxy-urs-12-ene-28-oic acid	D	2013	harvest	Royal Gala	17	2.97	10.6	17_21775385
p-coumaroyloxy-dihydroxy-urs-12-ene-28-oic acid	D	2013	harvest	Granny Smith	1	3.12	11.1	1_17233926
p-coumaroyloxy-dihydroxy-urs-12-ene-28-oic acid	A	2013	storage	Granny Smith	3	3.63	12.6	3_10537812
p-coumaroyloxy-dihydroxy-urs-12-ene-28-oic acid	A	2013	harvest	Granny Smith	3	3.16	11.2	3_11572647
p-coumaroyloxy-dihydroxy-urs-12-ene-28-oic acid	D	2013	harvest	Royal Gala	4	2.52	9.1	4_18467352
p-coumaroyloxy-dihydroxy-urs-12-ene-28-oic acid	D	2013	storage	Royal Gala	6	2.96	10.4	6_25368141
p-coumaroyloxy-dihydroxy-urs-12-ene-28-oic acid	E	2013	storage	Royal Gala	6	3.31	11.6	6_25368141
p-coumaroyloxy-dihydroxy-urs-12-ene-28-oic acid	A	2013	harvest	Royal Gala	9	2.85	10.2	9_32698252
p-coumaroyloxy-hydroxy-urs-12-ene-28-oic acid	C	2013	storage	Royal Gala	3	2.81	9.9	3_1143761
p-coumaroyloxy-hydroxy-urs-12-ene-28-oic acid	A	2013	harvest	Granny Smith	5	2.66	9.6	5_25120947
p-coumaroyloxy-hydroxy-urs-12-ene-28-oic acid	B	2013	harvest	Granny Smith	5	3.07	10.9	5_25120947
p-coumaroyloxy-hydroxy-urs-12-ene-28-oic acid	C	2013	harvest	Granny Smith	5	2.45	8.8	5_24406956
p-coumaroyloxy-hydroxy-urs-12-ene-28-oic acid	A	2013	harvest	Royal Gala	9	3.01	10.7	9_32698252
p-coumaroyloxy-hydroxy-urs-12-ene-28-oic acid	A	2013	storage	Royal Gala	9	3.2	11.2	9_32698252
p-coumaroyloxy-hydroxy-urs-12-ene-28-oic acid	B	2013	storage	Royal Gala	9	3.74	13	9_24219741
p-coumaroyloxy-hydroxy-urs-12-ene-28-oic acid	B	2013	harvest	Royal Gala	9	5.02	17.3	9_24219741
p-coumaroyloxy-hydroxy-urs-12-ene-28-oic acid	C	2013	storage	Royal Gala	9	5.79	19.3	9_18520824
p-coumaroyloxy-hydroxy-urs-12-ene-28-oic acid	C	2013	harvest	Royal Gala	9	5.68	19.7	9_20030488
p-coumaroyloxy-hydroxy-urs-12-ene-28-oic acid	A	2013	harvest	Royal Gala	17	3.54	12.5	17_21775385
p-coumaroyloxy-hydroxy-urs-12-ene-28-oic acid	B	2013	storage	Granny Smith	17	2.91	10.2	17_10872135
p-coumaroyloxy-hydroxy-urs-12-ene-28-oic acid	B	2013	storage	Royal Gala	17	2.55	9	17_21775385
p-coumaroyloxy-hydroxy-urs-12-ene-28-oic acid	B	2013	harvest	Royal Gala	17	2.71	9.7	17_21775385
p-coumaroyloxy-hydroxy-urs-12-ene-28-oic acid	C	2013	harvest	Granny Smith	17	2.73	9.8	17_21756554
p-coumaroyloxy-hydroxy-urs-12-ene-28-oic acid	C	2013	storage	Granny Smith	17	3.95	13.7	17_6940495
trihydroxy-urs-12-ene-28-oic acid	A	2013	storage	Granny Smith	3	2.79	9.8	3_11572647
trihydroxy-urs-12-ene-28-oic acid	A	2013	harvest	Granny Smith	3	3.24	11.5	3_11572647
trihydroxy-urs-12-ene-28-oic acid	na	2015	Storage	Granny Smith	3	5.46	22.4	3_34437600
trihydroxy-urs-12-ene-28-oic acid	na	2015	Harvest	Granny Smith	3	4.18	15.1	3_9950780
trihydroxy-urs-12-ene-28-oic acid	A	2013	storage	Royal Gala	5	2.43	8.6	5_10289990
trihydroxy-urs-12-ene-28-oic acid	na	2015	Storage	Royal Gala	5	6.09	24.9	5_15472298
trihydroxy-urs-12-ene-28-oic acid	na	2015	Harvest	Royal Gala	5	3.65	13.3	5_34448952
trihydroxy-urs-12-ene-28-oic acid	na	2015	Harvest	Granny Smith	8	4.13	14.9	8_18192194
trihydroxy-urs-12-ene-28-oic acid	A	2013	storage	Granny Smith	16	2.57	9.1	16_248882
trihydroxy-urs-12-ene-28-oic acid	na	2015	Storage	Granny Smith	16	3.74	16	16_248882
trihydroxy-urs-12-ene-28-oic acid	A	2013	storage	Royal Gala	17	3.43	12	17_21775385

## Discussion

While the pharmaceutical properties of pentacyclic triterpenes have been researched extensively^2^, no effort has been made to study the genetic control of the phenotypic variation of such compounds in apple fruit. Here, we discovered nine metabolite classes with multiple isoforms likely to belong to the chemical class of pentacyclic terpenes in the peel of apple fruit, with some variability measured in the progeny of a ‘Royal Gala’ x ‘Granny Smith’ cross. The metabolite concentrations varied between years and treatments (at harvest and after 12 weeks cold storage). The phenotypic correlations between fruit at harvest and after cold storage differed broadly, with some compounds having no correlation at all between the time points, indicating that complex physiological mechanisms influencing fruit ripeness may be at play. Our protocol involved storing fruit consistently for all collected fruit of the segregating progeny, however, it is likely that genetic variability exists for how fruit ripen. In fact, both parents have a contrasting physiology, with ‘Granny Smith’ requiring cold treatment to ripen while ‘Royal Gala’ ripens at warmer temperatures^27^.

We hypothesized that plant phytochemical composition can be improved by selective breeding assisted by genome information, alongside other horticultural traits such as taste, yield and disease resistance. There have been several examples of genetic studies of health targeted compounds in apple, such as vitamin C and polyphenol^28–30^, with the goal of developing genetic markers for selecting for higher concentrations of these health-related compounds. This is the first report of QTLs for pentacyclic triterpenes in apple, and only the second in fruit and nut species since QTLs for such compounds were reported for coconut^31^. Our results indicate that the genetic control of these compounds is polygenic, i.e. linked to many loci, each explaining a low proportion of the phenotypic variance. This could be due to ‘Royal Gala’ and ‘Granny Smith’ not being the best combination of parents to maximise segregation of pentacyclic triterpene concentration in their progeny, as this population was originally set up for its diversity in aromatic volatile composition^23^. Only QTLs on LG3, LG5 and LG16 showed some stability between years. Again, this could be due to variability in the physiological state of the fruit samples. We tried to mitigate variability in fruit maturity and ripening state by collecting fruit with similar starch indices and by storing them for 12 weeks, however, genetic differences accounting for differences in fruit maturity and ripening complicates the study of such sensitive compounds as pentacyclic triterpenes. Interestingly, none of the QTLs detected for pentacyclic triterpenes (both stable and non-stable between years) co-located with loci known to be involved in fruit ripening in apple, such as *ACO1* and *ACS1* on LG10 and LG15^32^.

Stable QTLs on LG3, LG5 and LG16 could be good candidates to develop markers for marker-assisted selection (MAS). Nevertheless, the small variance explained by these QTLs means that they are unlikely to be picked up by apple breeders for MAS. Instead, we would recommend the use of genomic selection^33^ for such polygenic traits.

## Conclusion

We have provided evidence that pentacyclic triterpene compounds can segregate in apple breeding crosses and that the concentration of these compounds is genetically controlled by many loci of small effect. This indicates that genomic information may need to be used in the future for breeding healthier apples.

## Supplementary information

Supplemental Material 1: Phenotypic variability for triterpene compounds in the ‘Royal Gala’ x ‘Granny Smith’ segregating population.

Supplemental Material 2: Chemical compounds detected by UHPLC in the progeny of the ‘Royal Gala’ x ‘Granny Smith’ population. m/z: mass (M-H-).

Supplemental Material 3: Triterpene compound correlations in the ‘Royal Gala’ x ‘Granny Smith’ segregating population.
